# Genome-wide analysis of basic helix–loop–helix (bHLH) transcription factors in *Aquilaria sinensis*

**DOI:** 10.1038/s41598-022-10785-w

**Published:** 2022-05-03

**Authors:** Pei-Wen Sun, Zhi-Hui Gao, Fei-Fei Lv, Cui-Cui Yu, Yue Jin, Yan-Hong Xu, Jian-He Wei

**Affiliations:** 1 Key Laboratory of Bioactive Substances and Resources Utilization of Chinese Herbal Medicine, Ministry of Education and National Engineering Laboratory for Breeding of Endangered Medicinal Materials, Institute of Medicinal Plant Development, Chinese Academy of Medical Sciences and Peking Union Medical College, Beijing, 100193 China; 2 Hainan Provincial Key Laboratory of Resources Conservation and Development of Southern Medicine and Key Laboratory of State Administration of Traditional Chinese Medicine for Agarwood Sustainable Utilization, Hainan Branch of the Institute of Medicinal Plant Development, Chinese Academy of Medical Sciences and Peking Union Medical College, Haikou, 570311 China

**Keywords:** Genetics, Plant sciences

## Abstract

The basic helix–loop–helix (bHLH) transcription factors are involved in several biological processes both in plant development and stress responses. Agarwood, a major active and economical product, is only induced and accumulated when the roots, stems, or branches are wounded in *Aquilaria sinensis*. Although genome-wide comprehensive analyses of the bHLH family have been identified in many plants, no systematic study of the genes in this family has been conducted in *A. sinensis*. In this study, 105 *bHLH* genes were identified in *A. sinensis* through genome-wide analysis and named according to their chromosomal locations. Based on a phylogenetic tree, AsbHLH family proteins were classified into 18 subfamilies. Most of them were distributed on eight chromosomes, with the exception of two genes. Based on the tissue-specific expression characteristics and expression patterns in response to methyl jasmonate (MeJA) treatment, seven *AsbHLH* genes were likely involved in wound-induced agarwood formation. The results provide comprehensive information on AsbHLHs that can be used to elucidate the molecular functions and physiological roles of these proteins in *A. sinensis*.

## Introduction

Agarwood is a precious fragrant natural product with high commercial and medicinal value. Agarwood has been widely used as a traditional medicine for tranquilizing and reducing excitement for centuries in China. It is also used in fragrances, incense, and aromatherapy. *Aquilaria sinensis* (Lour.) Gilg is an important agarwood-producing species, and it is the only certified medicinal source of agarwood in China^1^. It is widely distributed in Southern China in provinces such as Hainan, Fujian, Yunnan, and Guangdong^2^. Natural *Aquilaria* forests are severely endangered because of the overexploitation of wild resources and indiscriminate felling. All species of *Aquilaria* spp. are currently listed in Appendix II of the Convention on International Trade in Endangered Species of Wild Fauna and Flora^3^. Agarwood production is sustainable in South China and many Southeast Asian countries because of the large-scale cultivation and planting of trees in these regions^4^. However, the limited knowledge of the mechanism of agarwood induction limits increases in agarwood production. Agarwood is formed only after the trunk, root, or branch of *Aquilaria* trees is wounded^5^. Enhancing our understanding of the regulation and molecular mechanism of agarwood formation will shed light on the relationship between the stress responses of plants and agarwood induction. Here, we conducted a transcriptome and genome analysis of *A. sinensis* to identify and classify members of a major class of wound-related genes.

Transcription factors (TFs) play key roles in stress-related regulation networks and signal pathways. Basic helix–loop–helix (bHLH) TFs constitute a large superfamily, members of which have been identified in all eukaryotes^6–8^. In plants, the bHLH superfamily is the second largest family of TFs^9^. The bHLH TFs are named for their highly conserved alkaline/helix–loop–helix domains^10,11^, including a basic region and an HLH region^12^. The basic region is located at the N-terminus of the domain, which consists of approximately 17 amino acids and exhibits DNA-binding activity^13^. The HLH region is located at the C-terminal end and includes two amphipathic α-helices separated by a variable loop, which participates in interactions with proteins^13,14^. Outside of the two conserved regions, the rest of the sequences are vastly divergent^15^. Much more research on the bHLH family has been conducted in animals than in plants^12^. The bHLH TFs in animals were previously divided into six subgroups (A to F)^13,16^. Most bHLH TFs in plants are similar to animal Group B in their structural features, which can also be combined with E-box sequences^17^. However, their classification in plants is different and remains unclear. Based on a phylogenic tree in *Arabidopsis*, AtbHLHs were divided into 21 subfamilies^18–20^. In rice, 167 members were identified and divided into 22 subfamilies^21^. The genomes of an increasing number of plants have been sequenced, and several bHLH families were identified and studied. For example, the genomes of tomato, potato, maize, and peanut contain 159, 124, 208, and 261 bHLH genes, which were divided into 21, 15, 18, and 19 subfamilies, respectively^11,22–24^.

In plants, bHLH factors are involved in the regulation of plant development and the responses to environmental stress^7,8,17,19,25,26^. Lc (L-myc), which was identified as a bHLH protein in maize, regulates the biosynthesis of anthocyanins^27^. The bHLH protein PIL5 plays a key role in phytochrome-mediated seed germination in *Arabidopsis*^28^. In addition, members of the bHLH family in *Arabidopsis* have been reported to function in the establishment of the epidermal cells of roots and aerial tissues^29^. *Arabidopsis* fend off insect attacks through the production of toxic metabolites, such as glucosinolates (GSs). The bHLH TFs MYC2, MYC3, and MYC4 play crucial roles in the regulation of GS biosynthesis^30^. In *A. sinensis*, AsMYC2 (AsbHLH7) was found to participate in the regulation of agarwood sesquiterpene biosynthesis by controlling the expression of the sesquiterpene biosynthesis gene *ASS1*^31^. Although we previously sequenced the whole genome of *A. sinensis*, bHLH TFs have not yet been systematically characterized and analyzed. Here, a total of 105 *AsbHLH* genes were identified, and a series of genome-wide analyses were conducted, including analyses of gene structure, phylogenetic relationships, chromosome distribution, and expression patterns. Additionally, we identified several candidate genes that likely participate in the process of agarwood formation. This study provides important insights that will aid future research on the bHLH family in *A. sinensis*.

## Results

### Identification and characterization of bHLH proteins in *A.sinensis*

**Figure 1:**
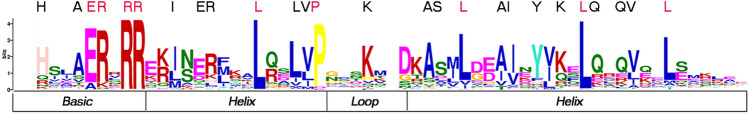
Conserved motif analysis of the bHLH domain in *A. sinensis*. The height of each amino acid represents the degree of conservation at the position. The amino acids with a conservation ratio more than 50% were identified with capital letters. And amino acids with greater than 75% identity were shown in red.

A search of the *A. sinensis* genome database revealed 105 putative *AsbHLH* genes, which were named according to their chromosomal location. The ORF sequence length of *AsbHLHs* varied from 276 bp (*AsbHLH96*) to 2151 bp (*AsbHLH91*), and the length of *A. sinensis* bHLH proteins ranged from 91 (AsbHLH96) to 716 (AsbHLH91) amino acids. The molecular masses of the bHLH proteins were predicted to range from 10.34 to 76.74 kDa, and the theoretical isoelectric points were predicted to range from 4.81 (AsbHLH15) to 9.81 (AsbHLH10) (Supplementary Table S1). Conserved amino acid residues in the AsbHLH protein was examined by multiple sequence alignment of their basic helix–loop–helix domain. Each AsbHLH protein contains four conserved regions, including the basic region, two helixes, and a loop region. However, there was no basic region in AsbHLH95, AsbHLH96 and AsbHLH97. The bHLH domain alignment showed that 26 amino acid residues were highly conserved, with a consensus of over 50% ratio (Fig. 1). Among these 26 residues, nine were conserved with a ratio of more than 75%, which were distributed in basic regions, the first helix region, and the second helix region.

**Figure 2:**
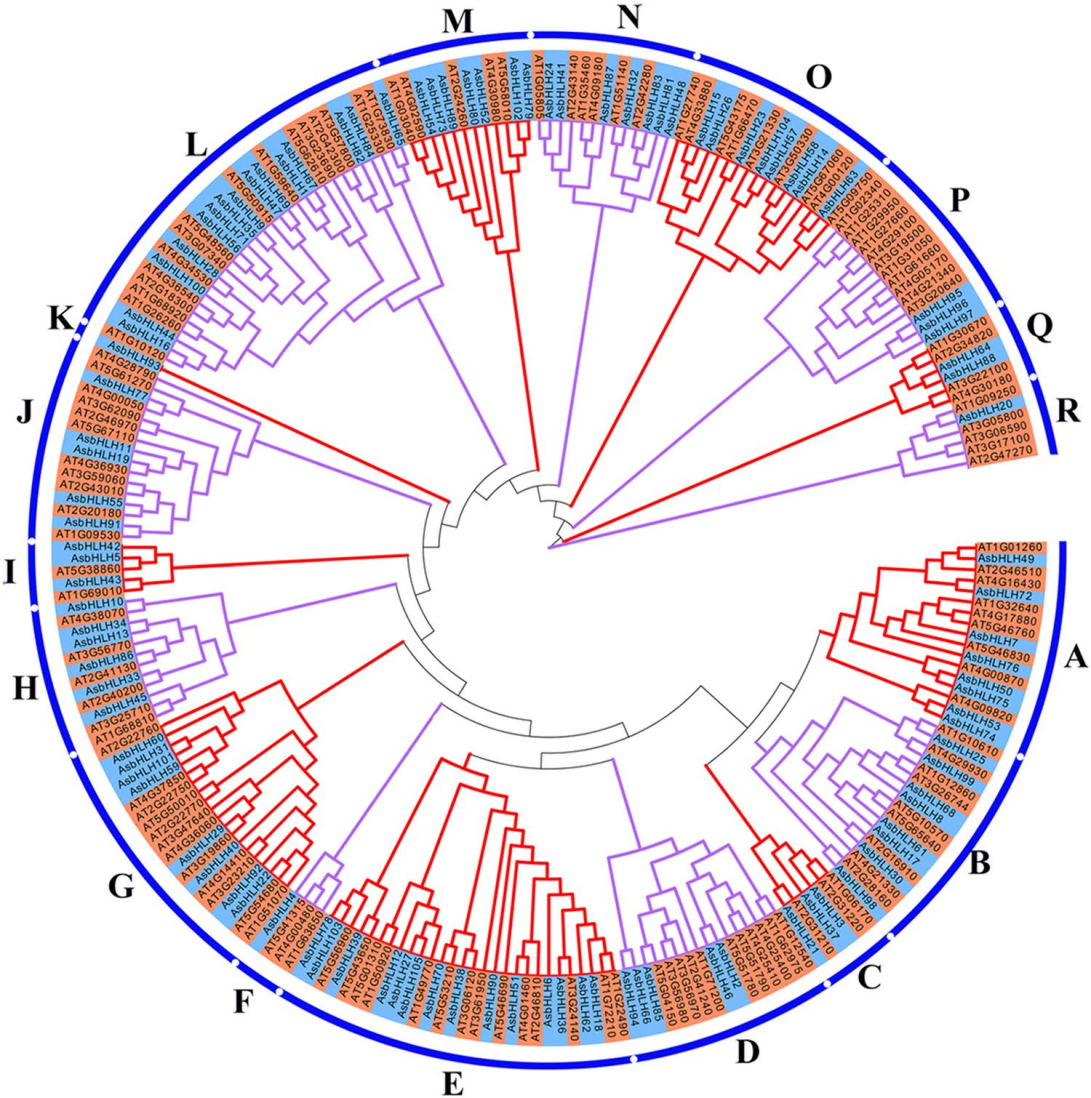
Phylogenetic analysis of bHLH proteins in *Arabidopsis* and *A. sinensis*. The Neighbor-joining tree was constructed using MEGA 5.0 software with 1000 bootstrap replications. The light blue represents proteins from *A. sinensis*, and the orange represents *Arabidopsis* bHLH proteins.

Evolutionary relationships within the AsbHLH members were analyzed, and a phylogenetic tree was constructed by aligning the AsbHLH proteins with the bHLH members from *Arabidopsis*^20^. AsbHLH family proteins were classified into 18 subfamilies (named A to R) (Fig. 2). The largest group in *A. sinensis* was subfamily L with 31 members, and subfamily K was the smallest, with only one protein from *Aquilaria* (AsbHLH93). Additionally, the numbers of *AsbHLH* genes within groups A, B, C, E, H, I, L, M, N and O were similar to that in *Arabidopsis*. The apparent differences in the number of genes were observed within groups D, P, and Q, and the number of *A. sinensis* was presented as half or less. In groups F and R, there was only one AsbHLH protein but with 3 and 5 AtbHLH members, respectively. Thus, interspecific divergence of the bHLH family between *A. sinensis* and *Arabidopsis* was obvious. To understand the subcellular localization of AsbHLHs, LOCALIZER was used to determine the predicted localization in plant cells. Most of the bHLHs were located in nucleus with a ratio of 88.57%, whereas only two and four proteins were predicted to be localized in the chloroplast and mitochondria, respectively (Supplementary Figure S1; Table S1). There were three proteins that were predicted to be localized to the nucleus and with transit peptides. AsbHLH93 and AsbHLH94 were predicted to be localized in the nucleus and mitochondria. AsbHLH10 was predicted to be localized in the nucleus and chloroplast.

### Gene structure and motif analysis

**Figure 3:**
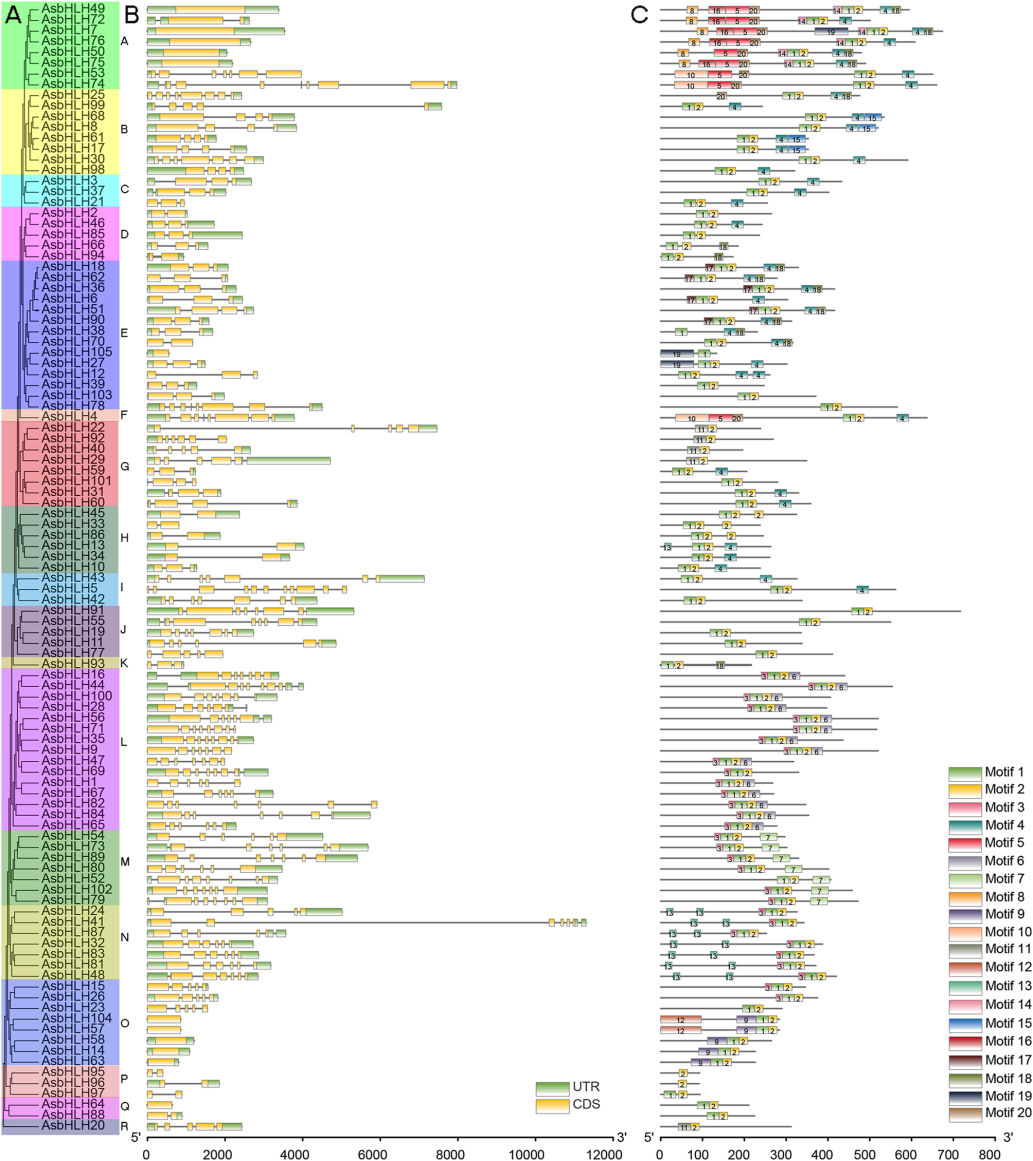
Analysis of gene structures and conserved motifs of AsbHLH members. (**A**) The phylogenetic relationship of AsbHLHs. (**B**) Exon–intron structure of *AsbHLH*s. The green boxes, yellow boxes and black lines represent untranslated 5′- and 3′-regions, exons and introns, respectively. (**C**) Motif analysis of the AsbHLH proteins. Twenty predicted motifs are displayed in different colored boxes. For the information of motif details refer to Supplementary Table S2. The size of the structures and motifs can be estimated by the scale at bottom.

Analysis of the gene structure within the *AsbHLH* genes can provide insight into the evolutionary relationships among gene families. The number of introns varies from 0 to 10 (Fig. 3B; Supplementary Table S1). Among the 105 *AsbHLH* genes, 93 genes were owner of the introns, and 12 genes were intron-less. Most members in this gene family had 1–7 introns. The intron distribution of genes within the subfamilies differed. The gene members of subfamily N contained 4–7 introns. As the two groups with the most members in this family, the genes in group D had 1–2 introns, and the introns of *AsbHLH* genes in group L ranged from 5 to 9. These results demonstrated that genes from the same subfamily have similar intron–exon compositions.

To identify and analyze the conserved motifs of AsbHLH proteins, a schematic was constructed using the MEME program. A total of 20 conserved motifs were identified (Fig. 3C; Supplementary Table S2). The length of 20 conserved motifs varied from 15 to 100 residues, and the numbers of conserved motif within the protein structures ranged from 1 to 10. Motif 1 and motif 2, located in bHLH domains, were distributed in almost all AsbHLH proteins. AsbHLH105 did not contain the conserved motif 2, and motif 1 was absent in the seven proteins (AsbHLH 20, -22, -29, -40, -92, -95 and -96). In a general, a motif usually appears only once, but we found that some motifs appeared twice in this analysis. In group N, motif 13 occurred twice in all members. Motif 2 occurred twice in some group H members (AsbHLH33, -45, and -86). The bHLH proteins in adjacent clades of the phylogenetic trees had the same or similar motif structure^11^. For example, members in group C had the same motif compositions. In group L, most members contained 4 motifs, motif 1, 2, 3, and 6, except for AsbHLH69. Aside from motifs 1, 2, and 7, proteins in group M also contained motif 3, but AsbHLH52 did not contain motif 3. We noticed that AsbHLH7 was special in group A. Compared with other members of the same group (AsbHLH49, -72, -76, -50, -75), it possesses a motif 19. In addition, their gene structures and motif compositions are similar (Fig. 3). Previous studies have shown that AsbHLH7 was involved in the synthesis of agarwood sesquiterpenes^31^. Moreover, proteins shared the similar structures in the same group may have similar functions. Thus, the gene structures and conserved motif analysis of AsbHLH members can provide new insights and clues for their evolutionary relationships and biological functions.

### Chromosomal location

**Figure 4:**
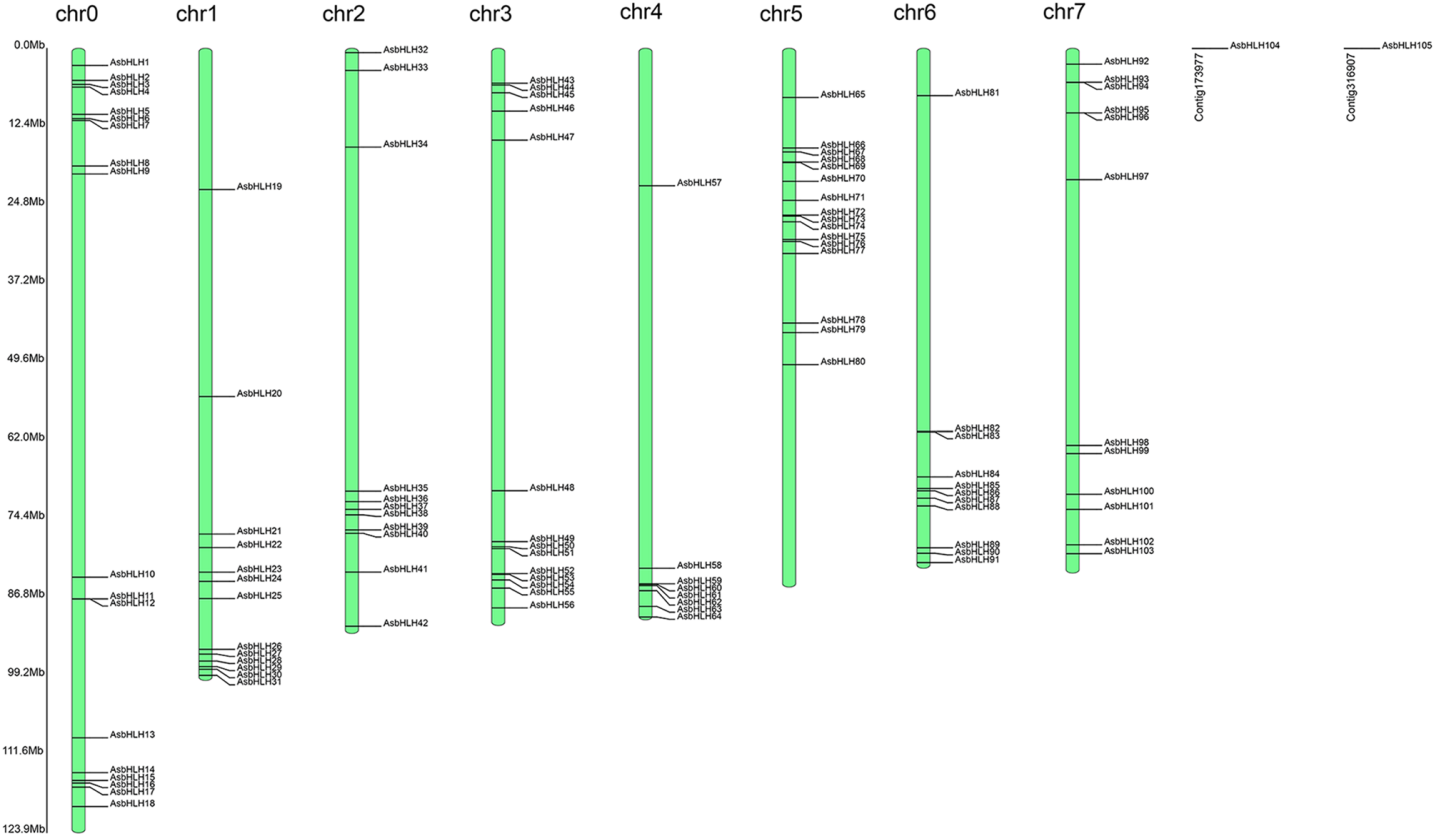
Distribution and localization of AsbHLHs on the *A.sinensis* chromosomes.

To characterize the chromosomal location of *AsbHLH* genes, the 105 members were mapped on the *A. sinensis* chromosomes based on the gene annotation information (Fig. 4). Except for *AsbHLH104* and *AsbHLH105*, the remaining 103 *AsbHLH* genes were irregularly localized on the eight chromosomes*.* Chromosome 0 (Chr0) contained the largest number (18) of *bHLH* genes, followed by Chr5, Chr3, Chr1, Chr7, Chr2 and Chr6; and Chr4 contained the least number (8) of genes. In addition, we found that the length of chromosomes differed. The arm of Chr0 was the longest, and Chr6 was the shortest chromosome. This indicated that the distribution of *AsbHLH* genes was not correlated with the length of Chromosomes.

### The ***AsbHLHs*** expression pattern in various tissues

**Figure 5:**
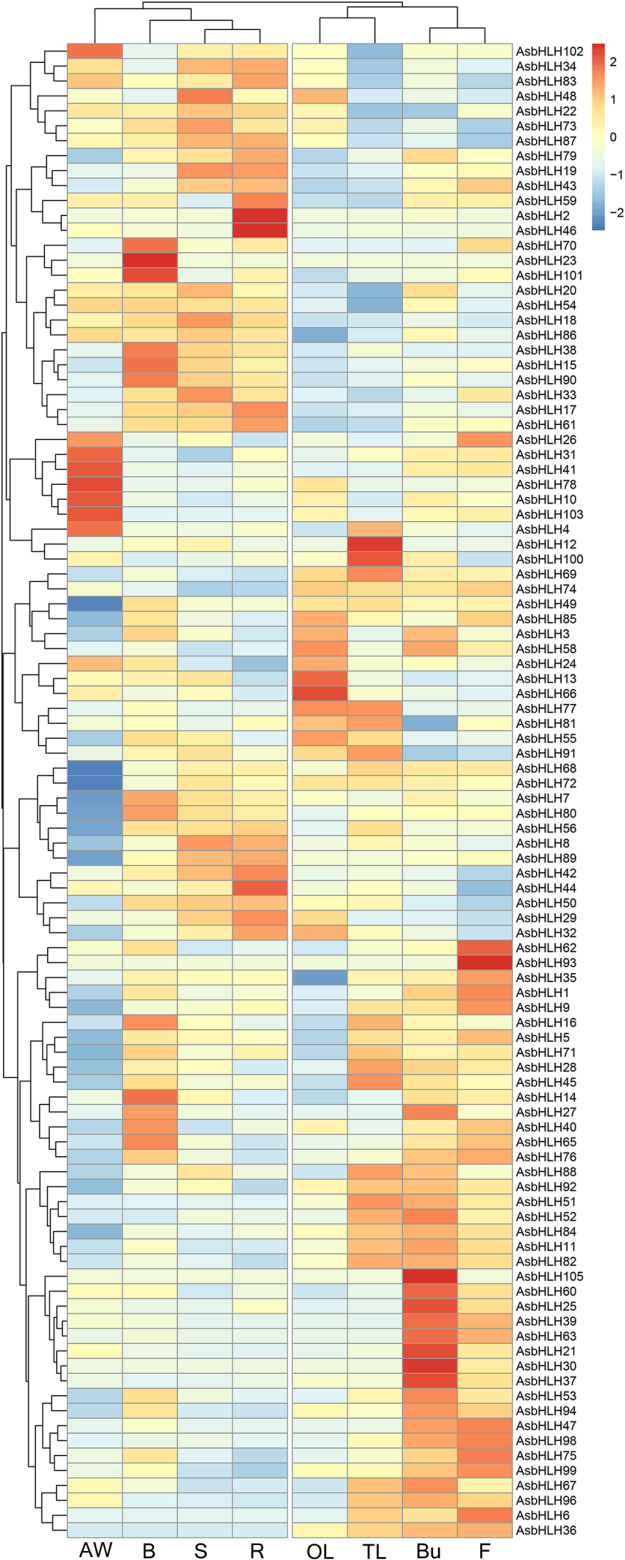
Expression profile of AsbHLH genes in different tissues. The color scale is displayed at the right. The high expression levels were colored red while low expression levels were shown in blue. (*AW* Agarwood, *B* branch, *S* stem, *R* root, *OL* old-leaf, *TL* tender-leaf, *Bu* bud, *F* flower).

The expression patterns of *AsbHLH* genes in various tissues (including agarwood, branch, stem, root, old-leaf, tender-leaf, bud and flower tissues) were characterized, and the expression heatmap were clustered based on the RPKM values (Fig. 5; Supplementary Table S3). According to the heatmap, the expression characteristics of *AsbHLHs* in these eight tissues were clearly divided into two groups. One group includes agarwood, roots, stems and branches; and the other group contains the remaining tissues, including tender leaves, old leaves, flower and bud tissues. Compared with the differential expression data in the eight tissues, 47 genes were expressed in agarwood, and seven (*AsbHLH 4, -10, -26, -31, -41, -78* and *-103*) were specifically highly expressed in agarwood layers, which were speculated to be related to the formation of agarwood. In contrast, some genes, such as *AsbHLH2, -17, -19, -38, -46, -61* and *-90*, were only highly expressed in roots, stems and/or branches, and were expressed at very low levels in the agarwood layer. These genes may also be related to the process of agarwood formation. In addition, five genes in tender-leaf and old-leaf tissues and11 genes in flower and bud tissues showed high tissue -specific expression. This suggests that these genes may be involved in the leaf and flower development.

To further confirm the expression patterns of *AsbHLH* genes in different tissues, we examined the transcriptional levels of nine genes in five different tissues (including agarwood, root, stem, branch and tender-leaf) by qRT-PCR. All genes were expressed in at least one of the tissues. *AsbHLH4* and *AsbHLH103* were highly expressed in agarwood, but low in other tissues. *AsbHLH2* was mainly expressed in root, and *AsbHLH 90* was highly expressed in branch. And *AsbHLH18* was preferentially expressed in stem, root and agarwood. Besides, *AsbHLH77* was only highly expressed in leaf, indicating its function in development. The characteristic of *AsbHLH54* was not special with expression in each tissue (Figure S2). Altogether, the results indicated that the expression pattern of the nine *AsbHLH* genes was roughly consistent with the trend in transcriptome data. And these genes with expression in agarwood and agarwood formation tissues could be potential candidates involved in agarwood production.

### Expression of ***AsbHLHs*** in response to MeJA treatment

**Figure 6:**
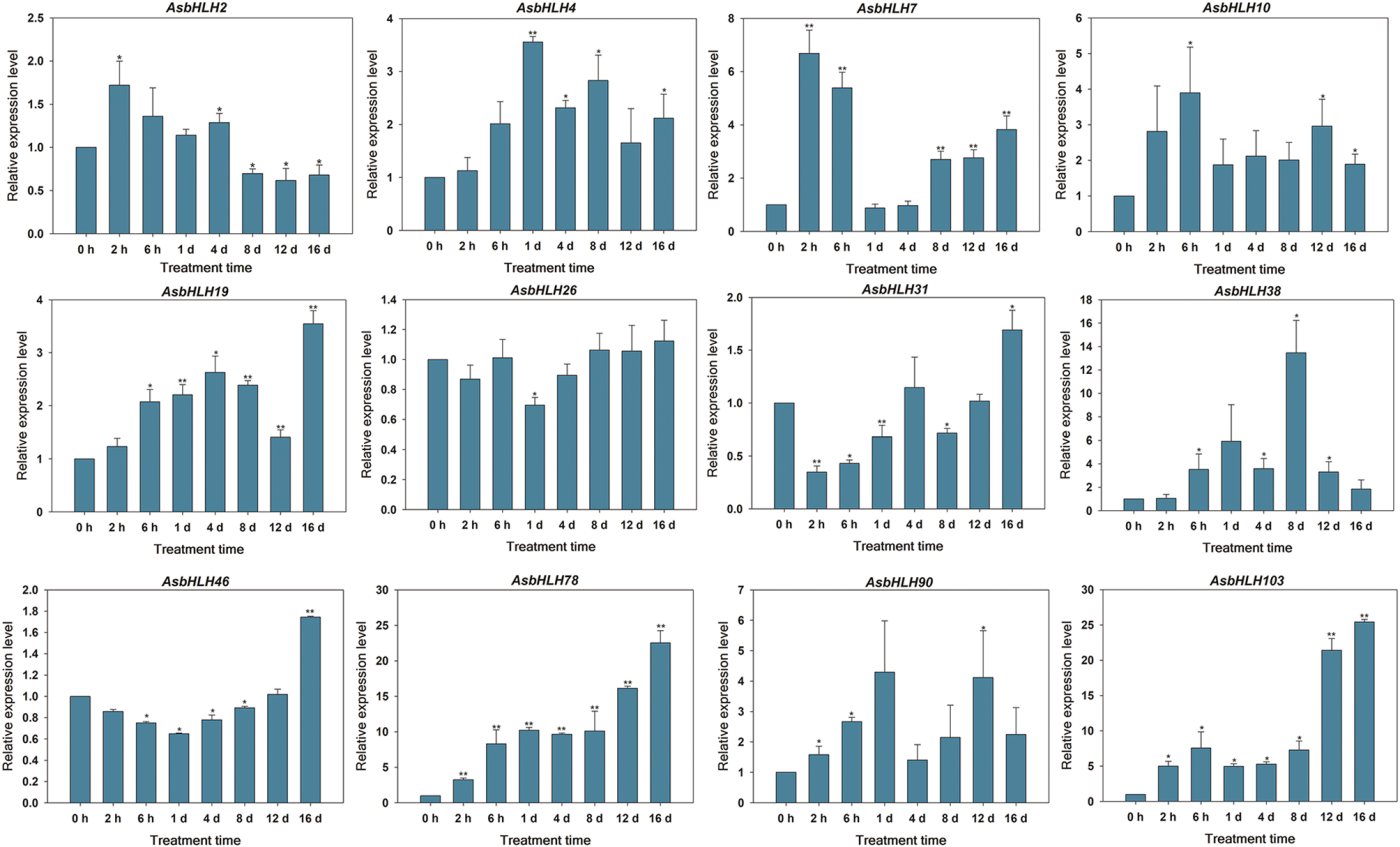
The relative expression levels of the 12 *AsbHLH* genes under MeJA treatments. *A.sinensis* calluses were treated with 100 µM MeJA for different times (0 h, 2 h, 6 h, 1 d, 4 d, 8 d, 12 d and 16 d ). The expression levels of *AsbHLH* genes at different times were compared with that at 0 h and *GADPH* was used as an internal control. Data are means (± SE) of three independent biological replicates. Asterisks indicate significant differences between each treatment point and controls (0-h) (* p < 0.05, ** p < 0.01, student’s t-tests).

MeJA has been reported to play an important role in the signaling pathway involved in agarwood formation^32^. To further examine the expression level of *AsbHLH* genes in response to MeJA treatment, qRT-PCR experiments were performed (Fig. 6). A total of 12 *AsbHLH* genes (*AsbHLH2, -4, -7, -10, -19, -26, -31, -38, -46, -78, -90,* and *-103*) were selected from the 105 AsbHLH genes based on their expression characteristics in various tissues. Under MeJA treatment, eight *bHLH* were significantly up-regulated, and the expression of another four genes varied little. *AsbHLH38, AsbHLH78,* and *AsbHLH103* were more sensitive to MeJA treatment and were notably up-regulated (> 10-fold). *AsbHLH4, AsbHLH7, AsbHLH10, AsbHLH19,* and *AsbHLH90* were slightly less up-regulated than *AsbHLH38, AsbHLH78,* and *AsbHLH103*. The eight up-regulated AsbHLH genes showed different expression patterns. *AsbHLH7* and *AsbHLH90* exhibited induced expression in an M-type, and the 4th day as the inflection point. Differently, the expressions of *AsbHLH19, AsbHLH78* and *AsbHLH103* generally increased gradually with the prolongation of MeJA induction. However, the expression level of *AsbHLH4, AsbHLH10* and *AsbHLH38* gradually increased to the peak and then decreased in the later period. These genes exhibited characteristics of inducible expression during the MeJA induction period. In comparison, the expression of *AsbHLH2, AsbHLH26, AsbHLH31,* and *AsbHLH46* did not change significantly in response to MeJA induction. Variation in the expression levels of the four *bHLH* genes was relatively low (< 2-fold). Previously, AsbHLH7 was found to participate in the regulation of agarwood sesquiterpene biosynthesis^31^. Thus, the remaining seven up-regulated genes (*AsbHLH4, -10, -19, -38, -78, -90* and *-103*) might contribute to the JA signaling pathway, and they might be involved in agarwood formation.

## Materials and methods

### Identification of the bHLH family members in *A. sinensis* genome

The sequence data of *A. sinensis* were available in the NCBI database (BioProject ID: PRJNA524272). The iTAK program (http://bioinfo.bti.cornell.edu/tool/itak) was used to characterize the bHLH TFs^33,34^. The HMMER3.1 program (http://hmmer.janelia.org) was used to identify integrated bHLH domains by comparing the plant protein sequences against the Pfam database (http://pfam.xfam.org). Default parameters were used, and the e-value cutoff was set to 0.001. All AsbHLH proteins were further examined and corrected with BLAST analysis and the Conserved Domain database (http://www.ncbi.nlm.nih.gov/cdd/). *Arabidopsis* bHLH sequences were downloaded from The Arabidopsis Information Resource (TAIR; http://www.arabidopsis.org/). Additionally, the Compute pI/Mw tool on the ExPASy server (http://web.expasy.org/compute_pi/) was used to predict the theoretical isoelectric point (pI) and the molecular weight (Mw) of the AsbHLH proteins. LOCALIZER (http://localizer.csiro.au/) was used to predict the subcellular localization of AsbHLH proteins^35^.

### Gene structure and protein motif analysis

Gene Structure Display Sever (http://gsds.cbi.pku.edu.cn) was used to analyze the exon–intron structures of each predicted AsbHLH. MEME software (http://meme-suite.org/tools/meme) was used to analyze the conserved protein motifs in AsbHLH proteins using the full-length protein sequences.

### Phylogenetic analysis and chromosomal location

Multiple domain alignments were performed using Clustal X 2.1 (http://www.clustal.org) analysis software. The phylogenetic tree was constructed in MEGA 5.0 (http://www.megasoftware.net) with full-length amino acid sequences of bHLH from *Arabidopsis*^20^ and *A. sinensis*. And the tree was built using the Neighbour-Joining method with 1000 bootstrap replications. The positional data of each *AsbHLH* genes on the chromosomes were obtained based on Hic (Chromosome conformation capture) result of *A. sinensis* genome.

### Tissue expression analysis

The expression pattern of *AsbHLH* genes in different tissues (agarwood, branch, stem, root, old-leaf, tender-leaf, bud, flower) was drawn using R script based on the normalized values (log2(RPKM + 1)) from RNA-seq data. The heatmap was normalized using the Z-score standardization method^36^.

### Plant materials and treatment conditions

*A. sinensis* (Lour.) Gilg was analyzed in this study and the formal identification of the plant materials was undertaken by Mr. J.H. Wei. We got the permission to collect the plant samples and all methods were performed in accordance with the relevant guidelines and regulations. The materials for RNA-seq are tissues from seven-year-old *A.sinensis* in Hainan, which belong to the Hainan Branch of the Institute of Medicinal Plant Development. The trees were treated using Whole-tree agarwood-inducing technique (Agar-Wit) to generate agarwood^36^. The old-leaf, tender-leaf, flower and bud from different developmental stages, the agarwood, stem, root and branch were collected separately to investigate the tissue expression patterns. The transcriptome sequencing libraries were generated using NEBNext^®^ Ultra™ RNA Library Prep Kit for Illumina^®^ (NEB, USA). The library preparations were sequenced on an Illumina Hiseq platform^36^. To further verify the expression patterns, the agarwood layer, root, stem, branch, tender-leaf were collected for qRT-PCR analysis.

The *A.sinensis* calli were induced from the fresh young stems of *A. sinensis* plantlets which were grown in the green house of IMPLAD. And they were grown in Murashige-Skoog (MS) medium with different combinations of auxins and cytokinins at 25 °C in dark. To investigate the expression patterns of *AsbHLH* genes under MeJA treatment, *A. sinensis* calli was grown in the medium with 100 μM MeJA for a range of times between 0 and 16 days (0 h, 2 h, 6 h, 1 day, 4 days, 8 days, 12 days and 16 days) for qRT-PCR analysis. All of the samples were collected and frozen in liquid nitrogen at the pointed time after treatment. All materials were stored at – 80 °C.There were three biological replicates for each sampled tissue.

### Total RNA isolation and qRT-PCR analysis

Total RNA was extracted from *A. sinensis* calli and tissues using a Total RNA extraction kit (Aidlab, China), supplemented with on-column DNA digestion per the manufacturer’s instructions. Single-stranded cDNA was synthesized from the total RNA using a PrimeScript™ RT reagent Kit with gDNA Eraser (Perfect Real Time; Takara, Japan) per the manufacturer’s protocols and subjected to qRT-PCR analysis using the LightCycler (Roche Diagnostics, Indianapolis, IN, USA). The relative expression level for each candidate genes under MeJA treatment was calculated using the 2^–ΔΔCq^ method and the 2^–ΔCq^ method for the tissue expression profile. *GADPH* was used as the internal reference gene. The primers used for qRT-PCR reactions were designed for the selected bHLH genes using Primer Premier 5.0 and listed in the Supplementary Table S4.

## Discussion

The bHLH family is a large group of TFs in plants that have been discovered and identified in multiple species. They have been found to play important roles in various biological processes of plant development and stress responses^19^. The present study is the first to systematically examine bHLH proteins in *A. sinensis*. It suggests that bHLH proteins may play important roles in agarwood formation and will aid the identification of bHLH proteins involved in this process.

The classification of bHLH proteins in plant is uncertain and differs in different species. The *bHLH* genes were previously divided into 21 subfamilies in *A. thaliana*^20^. To analyze the evolutionary relationships of *AsbHLH* genes, we constructed a phylogenetic tree by aligning the AsbHLH proteins with *Arabiopsis* bHLH members. The *AsbHLH* family genes were divided into 18 subfamilies (Fig. 2). With the similar method, the genes of this family were classified into 15 groups in *Solanum tuberosum*^11^, 15 in *Capsicum annuum*^37^, 17 in *Ginkgo biloba*^38^, and 19 in *Dracaena cambodiana*^39^. These classifications showed similarities and differences compared with the classification of these genes in *A. thaliana*. In general, genes with similar functions were clustered on the same branch. There were 36 AsbHLH members that were grouped with the bHLH proteins in *Arabidopsis*, which were predicted as homologous genes in *A. sinensis* and *A. thaliana* (Supplementary Figure S3). For example, in group A, AsbHLH 49 and AsbHLH 72 were orthologous to JASMONATE ASSOCIATED MYC2-LIKE2 (JAM2) (AT1G01260) and JAM3 (AT4G16430), respectively. JAMs negatively regulate jasmonate biosynthetic and JA-responsive genes^40^. Thus, we can surmise the two bHLH proteins in *A. sinensis* also participate in the regulation of JA responses. In *A. thaliana,* bHLH122 (AT1G51140) was strongly induced by drought, NaCl, and osmotic stress and functioned as a positive regulator of these signaling^41^. AsbHLH32, which is an ortholog of AtbHLH122, is highly expressed in roots and might be related to these multiple stresses. These findings can aid the prediction of functional genes in *A. sinensis*.

The conserved motif and gene structure analyses provide important information for resolving phylogenetic relationships. Most *AsbHLH* genes in the same subfamily shared similar motifs and exon–intron compositions (Fig. 3). Motifs 1 and 2, which are bHLH domains, were distributed in most AsbHLH proteins. Moreover, the composition of other motifs was unique, and motifs were conserved across subgroups. For example, motif 7 is unique in subgroup M, and motif 6 only appeared in the L group. Thus, these non-bHLH domains were necessary for the phylogenetic analysis. A similar phenomenon has been observed in other plants^42,43^. These results indicated that these characteristics have increased the diversity of AsbHLH proteins in different subgroups.

Members of the bHLH family in plants are involved in the regulation of plant development and responses to environmental stress^7,8,17,19,25,26^. In *A. sinensis*, agarwood is only produced in the roots, stems, and branches of *Aquilaria* trees after they are wounded^5^. The expression of AsbHLH in the eight tissues was divided into two groups. One group contains agarwood and its formation tissues including the roots, stems, and branches; and the other group includes the tender leaves, old leaves, flowers, and buds (Fig. 5). The former group was more closely related to the wound-induced formation of agarwood, which is the main concern of *A. sinensis*. Generally, genes related to agarwood formation are highly expressed in the former group, and genes with a high level of expression in the latter group are related to the physiological functions of non-agarwood production. For example, *AsbHLH77* was only highly expressed in leaves, and its homologous gene *PIF8* (AT4G00050) in *Arabidopsis* was involved in the phyA-mediated light responses^44^. This result suggests that *AsbHLH77* might be involved in the regulation of the light responses. AMS (AT2G16910) TF is a master regulator of sporopollenin biosynthesis, secretion, and pollen wall formation in *Arabidopsis*^45^. *AsbHLH30*, which is an ortholog of *AMS*, is highly expressed in buds and may be involved in pollen development. MeJA is an important signaling molecule that can induce agarwood sesquiterpene production and accumulation^32^. Interestingly, under MeJA treatment, the eight up-regulated AsbHLH genes responded at various times in a matter of 1 day up to multiple days later (Fig. 6). These results demonstrated that these AsbHLH genes might be play crucial roles in the JA signaling. Tissue expression analysis showed that *AsbHLH4, AsbHLH10, AsbHLH78* and *AsbHLH103* were only highly expressed in the agarwood layers (Fig. 5). Combined with the sensitive responses of these genes to MeJA treatment, it was suggested that these genes are stress-induced and may be involved in the formation of agarwood. In contrast, *AsbHLH7*, *AsbHLH 19, AsbHLH38, AsbHLH90* were highly expressed in healthy stems, roots or branches but barely expressed in agarwood layers (Fig. 5). Previously, AsbHLH7 was reported to mediate the regulation of agarwood sesquiterpene biosynthesis in *A. sinensis*^31^. Therefore, it was presumed that *AsbHLH 19*, *AsbHLH38*, *AsbHLH90* are also likely involved in the process of agarwood formation. These aforementioned findings may facilitate future research examining the function of *AsbHLHs* in the process of agarwood formation.

## Supplementary Information

Supplementary Information.
