# Supplementary material for: Genome-wide analysis of basic helix–loop–helix (bHLH) transcription factors in Aquilaria sinensis
Source: Sci Rep. 2022 May 3;12:7194. doi: 10.1038/s41598-022-10785-w (PMC9065063; doi:10.1038/s41598-022-10785-w)
Supplement: Supplementary file 1 — Supplementary Information. [file 41598_2022_10785_MOESM1_ESM.zip › Supplemntary material/Supplementary Figure S1-S3.docx]

Genome-wide analysis of basic helix-loop-helix (bHLH) transcription factors in *Aquilaria sinensis*

**Pei-Wen Sun^1^, Zhi-Hui Gao^1^, Fei-Fei Lv^2^, Cui-Cui Yu^1^, Yue Jin^1^, Yan-Hong Xu^1, *^, Jian-He Wei^1, 2, *^**

^1^ Key Laboratory of Bioactive Substances and Resources Utilization of Chinese Herbal Medicine, Ministry of Education and National Engineering Laboratory for Breeding of Endangered Medicinal Materials, Institute of Medicinal Plant Development, Chinese Academy of Medical Sciences and Peking Union Medical College, Beijing 100193, China.

^2^ Hainan Provincial Key Laboratory of Resources Conservation and Development of Southern Medicine and Key Laboratory of State Administration of Traditional Chinese Medicine for Agarwood Sustainable Utilization, Hainan Branch of the Institute of Medicinal Plant Development, Chinese Academy of Medical Sciences and Peking Union Medical College, Haikou 570311, China.

*** Correspondence:**Yan-Hong Xu (xuyanhong99@163.com) and Jian-He Wei (wjianh@263.net)


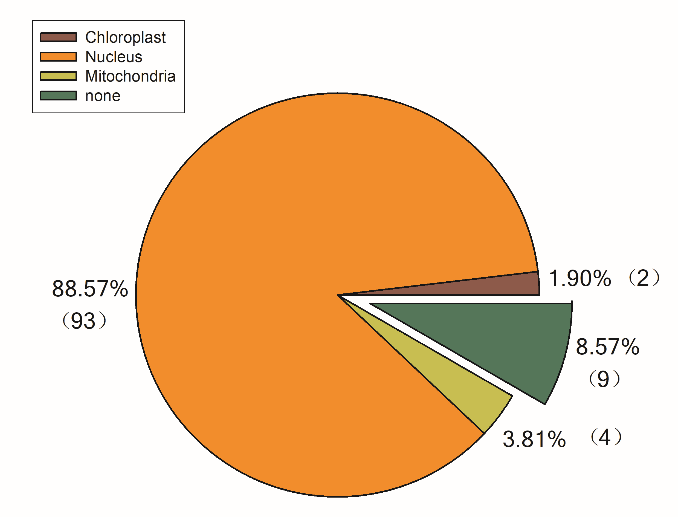


**Supplementary Figure S1** Prediction result of subcellular localization of AsbHLH gene family. The pie charts were created using the value of AsbHLH localization. The orange represents the nucleus localization; the brown represents the chloroplast localization; the yellow represents the mitochondria localization, and the dark green indicates that no positioning signal was detected.

(Note: There were three proteins that were predicted to be localized to the nucleus and with transit peptides. Two were predicted to be localized in the nucleus and mitochondria and the other in the nucleus and chloroplast.)


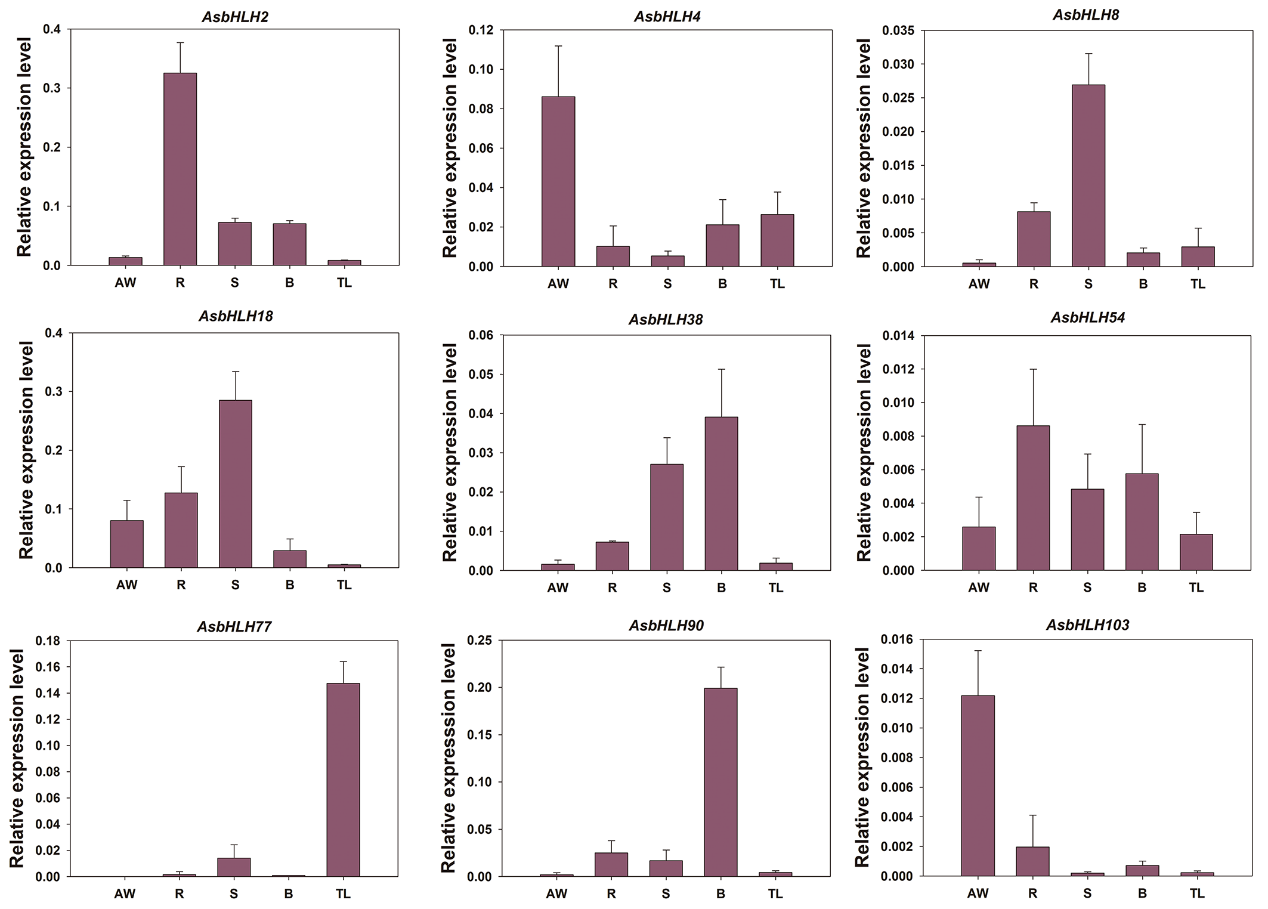
**Supplementary Figure S2** Expression analysis of *AsbHLH* genes in various tissues (agarwood, root, stem, branch and tender-leaf) by qRT-PCR. Data are means (±SE) of three independent biological replicates. The relative expression level was calculated by 2^–ΔCq^ method, and *GADPH* was used as the internal reference gene. (AW: Agarwood; R: Root; S: Stem; B: Branch; TL: Tender-leaf).


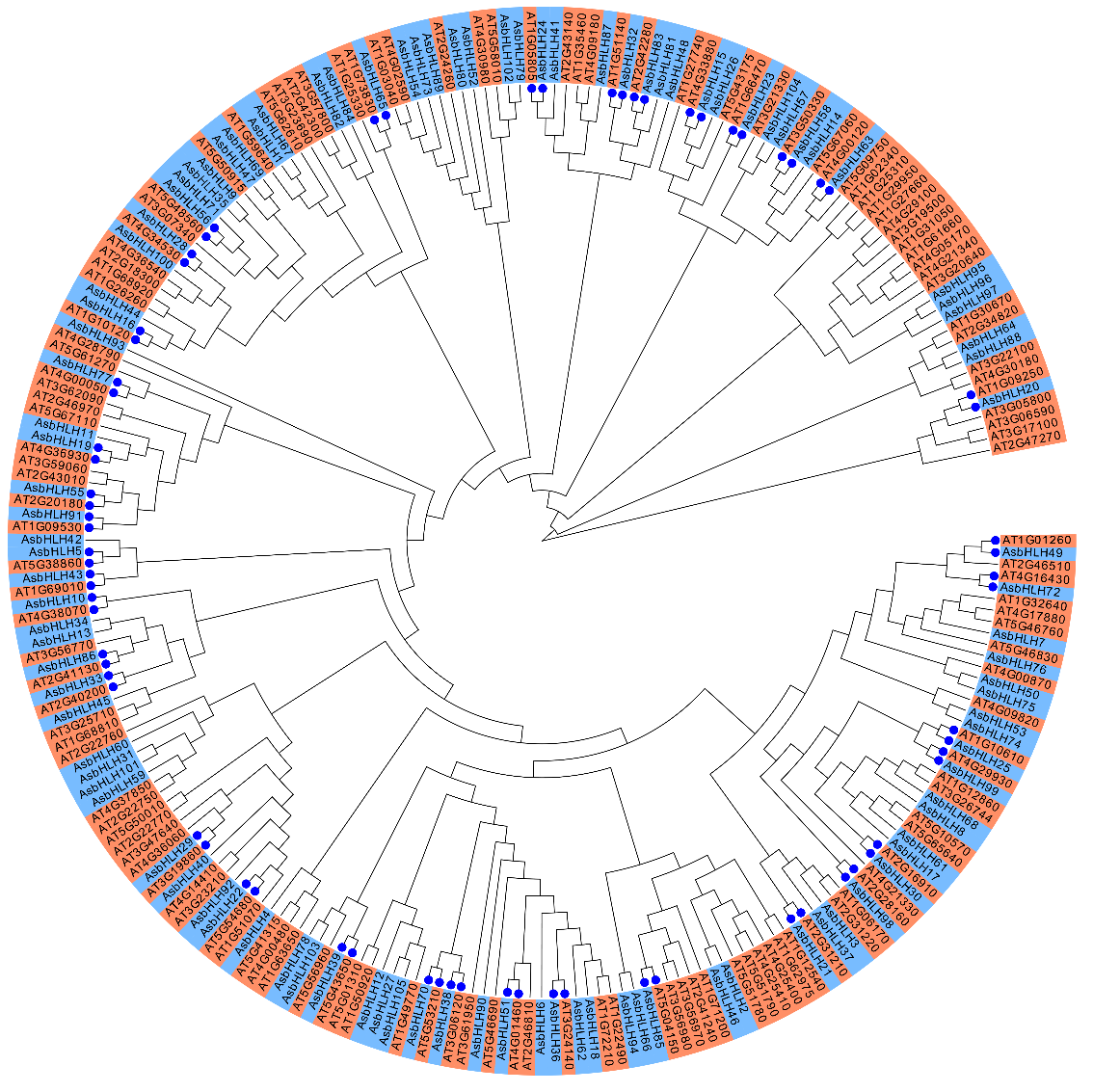


**Supplementary Figure S3** AsbHLHs tightly grouped with the AtbHLH proteins in the phylogenetic tree. The blue dots represent the tightly grouped AsbHLH proteins and AtbHLH proteins.
